# Fast Negative Feedback Enables Mammalian Auditory Nerve Fibers to Encode a Wide Dynamic Range of Sound Intensities

**DOI:** 10.1371/journal.pone.0032384

**Published:** 2012-03-08

**Authors:** Mark Ospeck

**Affiliations:** Independent, Boulder, Colorado, United States of America; University of Southern California, United States of America

## Abstract

Mammalian auditory nerve fibers (ANF) are remarkable for being able to encode a 40 dB, or hundred fold, range of sound pressure levels into their firing rate. Most of the fibers are very sensitive and raise their quiescent spike rate by a small amount for a faint sound at auditory threshold. Then as the sound intensity is increased, they slowly increase their spike rate, with some fibers going up as high as ∼300 Hz. In this way mammals are able to combine sensitivity and wide dynamic range. They are also able to discern sounds embedded within background noise. ANF receive efferent feedback, which suggests that the fibers are readjusted according to the background noise in order to maximize the information content of their auditory spike trains. Inner hair cells activate currents in the unmyelinated distal dendrites of ANF where sound intensity is rate-coded into action potentials. We model this spike generator compartment as an attenuator that employs fast negative feedback. Input current induces rapid and proportional leak currents. This way ANF are able to have a linear frequency to input current (f-I) curve that has a wide dynamic range. The ANF spike generator remains very sensitive to threshold currents, but efferent feedback is able to lower its gain in response to noise.

## Introduction

Mammals have a powerful cochlear amplifier and so are able to have very low auditory thresholds for detecting sound waves (∼0 dB SPL, corresponding to micro Pascal pressure fluctuations) [1]. But surprisingly, they are also able to distinguish variations in sound intensity at levels ∼70 dB above this sensory threshold (10^7^ fold power increase) [1], [2]. Adaptive processing of sound levels is known to occur throughout the auditory pathway, and there is evidence that it results in drawing auditory attention towards a high probability region of sound intensities [3]. Adaptive processing begins with the hair cells and auditory nerve fibers (ANF) at the periphery. There, a graded neurotransmitter signal from an inner hair cell (IHC) is first encoded into a spike train within a small compartment in the dendrite of an ANF. ANF “digitize” the information content of a sound wave into a series of parallel spike trains, with each fiber's output spike range limited to about 300 Hz. Most fibers are sensitive to very faint sounds, but at the same time still respond to a wide dynamic range of sound inputs. This contradiction is known as the dynamic range problem in mammalian hearing [2]. Essentially, the problem is how to account for a vast range of hearing in which a very sensitive mammalian hearing apparatus is nevertheless able to rate code sound intensity across a gigantic input power range.

Each inner hair cell (IHC) sends ∼20 ANF with different sensitivity thresholds to the cochlear nucleus. Most IHC have low thresholds (0–20 dB SPL) with high spontaneous firing rates of up to ∼100 Hz. The remaining ∼20% have high thresholds and low spontaneous firing rates (∼0 Hz) [1], [4]. Part of the dynamic range problem is no doubt solved by having different classes of nerve fibers with different sensitivity ranges. However, a typical ANF has a range of ∼40 dB between its threshold and its saturation. Accounting for this 10,000 fold input power range, or ∼100 times input current range, already presents a huge dynamic-range stretching problem for a small neuronal compartment's spike generator. There are two distinct kinds of spike generators, class 1 and class 2 excitable. Both are strongly nonlinear, turning on abruptly when a current threshold is passed [5]–[7]. Each is founded on its own distinct bifurcation—a mathematical classification of the underlying mechanism by which its resting state is destabilized in order to make an action potential [7]. For both types of generator, the sharp rise in spike rate occurring just above its current threshold eats up a large amount of its output spike rate range. Previously, negative feedback has been investigated as one likely means for slowing down a spike generator's initial rate of increase, specifically in the case of cortex pyramidal neurons [8]. This result has been mathematically generalized; it is a generic property of strongly nonlinear spike generators that negative feedback is able to linearize their firing frequency vs. input current (f-I curve), provided that their no-feedback f-I curve is sufficiently nonlinear [9]. Negative feedback is not the only method for linearizing an f-I curve, but noise and changes in the variance of an input signal can also do the trick [10], [11].

**Figure 1:**
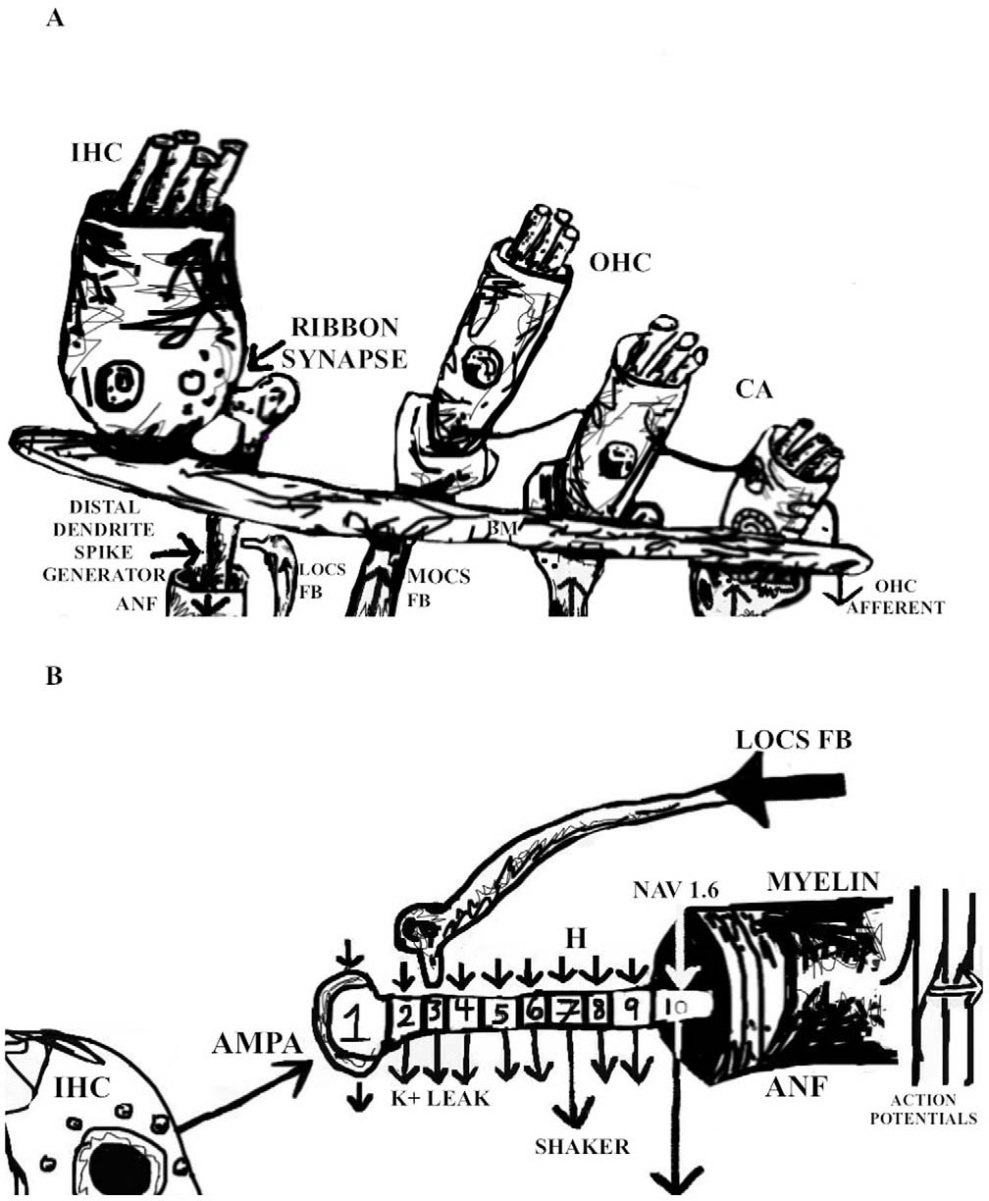
Cartoon of the mammal's auditory periphery. A. Outer hair cells (OHC) of the cochlear amplifier (CA) amplify the vibrations of a sound wave, increasing basilar membrane (BM) oscillation. Feedback from the medial olive (MOCS FB) controls OHC/CA gain. The amplified oscillations of OHC hair bundles are sensed by hair bundles of inner hair cells (IHC) and these cells then release neurotransmitter to the unmyelinated distal dendrites of auditory nerve fibers (ANF). Spike trains begin at the point close to where their myelination begins, with the lateral olive (LOCS FB) providing feedback to the ANF. OHC afferents report on the collective state of groups of OHC. B. Sketch of a 10 compartment model of the ANF distal dendrite from its synapse with an IHC to its heminode region where myelination begins. AMPA channels are the predominant glutamate-gated conductance at the synapse (compartment 1), while its heminode (compartment 10) has a high concentration of the fast sodium conductance NAV 1.6. ANF contain inactivating Shaker-type and non-inactivating Shaw-type K+ conductance, small amounts of Ca++ conductance and an inward leak, H-type conductance has been localized to the dendrite. Auditory action potentials are initiated in this unmyelinated distal dendrite which has a capacitance of only ∼1 pF [13], [14].

Spike generation in auditory nerve fibers has previously been mathematically modeled as a Poisson process [12]. But recently it has become clear that action potentials are first generated in the ANF dendrite (Fig. 1) [13]. So here we present a conductance-based model of the distal dendrite/encoding membrane region of an ANF. First we review what is known about the distal dendrite and how this relates to the dynamic range problem. Then we construct a model in which fast negative feedback is used to linearize the nerve fiber's f-I curve and to extend its dynamic range.

## Results

### Model

Below about 3 kHz, ANF phase lock their action potentials to the sound waves sensed by their respective IHC [1]. Here we consider only non phase-locking, higher frequency ANF, since the majority of experiments are done on such fibers from mice and rats [13]–[15]. In mouse and rat, the post-synaptic distal dendrite is less than a micron in diameter and it goes unmyelinated until reaching the heminode at the fenestratum (the window of the cochlea), a length of some 20 to 50 microns [13]. This combination of length scales and lack of myelination is curious considering that myelin was a vertebrate innovation that lengthened a neuron's space constant (the distance from a point maintained at a constant potential to a point where that potential decays e-fold, or by about 63%). The space constant is proportional to the square root of the axon radius divided by the square root of its membrane conductance per unit area [5]. In a myelinated axon it is typically maintained in a range from millimeters up to centimeters, with the gaps in myelination (the unmyelinated node of Ranvier spike repeaters) kept to less than one micron in length [6]. Inside the cochlea it seems that the ANF radii are intentionally kept low and membrane conductance high, so that the fibers maintain a short space constant calculated to be in the 100 micron range.

The following conductances are found in mammalian ANF. In mouse, NAV 1.6, a fast sodium (Na+) channel, typically found at nodes of Ranvier, has been localized to the distal dendrite's heminode region where myelination begins [13]. Shaker A-type conductance (an inactivating potassium channel that employs a blocking ball) accounts for about 60% of the potassium (K+) conductance in rat ANF [16]. Shaker is commonly used to space the interval between action potentials. Shaw-type, non-inactivating K+ conductance (the delayed rectifier part of an action potential generator) has been found in both rat and guinea pig ANF [16], [17]. So far, more specific experiments on rat dendrite reveal the presence of both high and low threshold K+ conductance [14]. ANF also contain ∼15 nS of H conductance, a monovalent cation channel responsible for a mostly Na+ inward leak current. Several nS of H have been localized to the distal dendrite in rat (reversal potential −45 mV in the dendrite) [14], [18]. H turns on slowly with hyperpolarization, but the dendrite's weakly voltage sensitive version (∼11 mV) has a very low half-on voltage, around −104 mV, that would appear to make it functionally irrelevant. This has led to the hypothesis that essential second messengers necessary for H activation are lost during experiments [14]. The dendrite's resting potential is −64 mV [14], and here we assume that significant amounts of H conductance remain on in the range just above it. AMPA conductance with a linear current-voltage relation and a reversal potential near 0 mV, is the predominant glutamate-gated conductance in rat [19]. Also, small amounts of calcium (Ca++) conductance have been localized to the dendrite whose input impedance in post natal rat is *Z_dc_*∼400 M Ohms, indicating that about 2.5 nS of conductance is on at rest [14]. The dendritic spike generator has a threshold of ∼−50 mV with a quantal EPSP, or “mini”, due to single vesicle release of ∼2.4 mV (rise time ∼1 ms, fall time ∼4 ms; corresponding mini current ∼40 pA EPSC with rise time ∼0.4 ms, fall time ∼1.2 ms; about 0.4 nS of AMPA conductance is turned on by the mini) [14]. During maximal IHC transmitter release, AMPA current can approach 800 pA [14], [19]. One unusual and important point is that the capacitance (*C*) of the dendritic spike generator compartment is only ∼1.3 pF. This is an order of magnitude smaller than the capacitance of a neuron soma where the typical spike generator is located [14], [6]. Evidently, *C* has been kept low so that relatively small currents are able to make large depolarizations. The time delay between current and voltage for a mini suggests that dendritic admittance *(1/Z)* is mostly capacitive in nature, and here a simple calculation agrees: *Z_mini_* = 2.4 mV/40 pA∼60 M Ohms, which is close to *Z_cap mini_*∼100 M Ohms = *1/(ω C)* = 1/(2π 10^3^ 1.3 10^−12^). It seems that for small kHz range currents (like a mini), the passive *RC* time constant *Z_dc_ C*∼0.5 msec, imposes a corner frequency of ∼300 Hz, which is the main cause of their attenuation.

The experimental results listed above suggest that the dendrite has been made to cross purposes, i.e. to be very sensitive with a low current threshold for spiking (low *C*), while at the same time to be used for attenuation (unmyelinated and leaky). At least for high frequencies, ANF are known to act as linear filters: signal gain imposed by the cochlear amplifier, and seen in the amplitude of the basilar membrane oscillation, is accurately represented by their spike rate [20]. How can a strongly nonlinear spike generator be made into a linear filter? Below we construct a 10 compartment model of the ANF dendrite with AMPA current input into compartment 1 and fast Na+ conductance in compartment 10 (the spike generator with a spike threshold of approximately −50 mV; Fig. 1). For a high frequency non phase-locking fiber AMPA input will be approximated by a square current pulse.

Here the basic idea is that in order to initiate action potentials AMPA input current must first pass through a cable with a variable input impedance. As AMPA current is increased, input impedance decreases, making it harder to spike. There are two basic ways that this kind of fast negative feedback can be made to work, either a first messenger approach where an increasing membrane potential directly turns on leak conductance, or where a second messenger such as Ca++ turns on leak. Both approaches can be made to work, although using a second messenger is easier. Another paper will investigate average membrane potential directly turning on leak conductance, but here we use the second messenger Ca++ to turn on leak.

Equations listing the currents into and out of the 10 compartments:
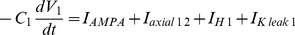
(1)

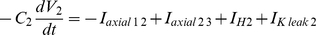
(2)

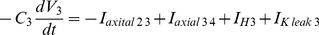
(3)

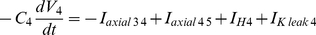
(4)

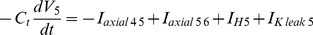
(5)

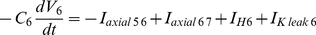
(6)

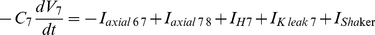
(7)

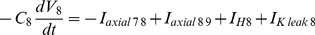
(8)

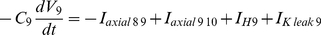
(9)

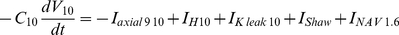
(10)The capacitance of each compartment *C_n_* is 0.15 pF. Outward current from each is by convention positive, and *I_axial n m_* represents the axial current out of compartment *n* into compartment *m*. These current-bookkeeping equations correspond to the first 10 of 17 differential equations in a Mathematica model of the dendrite, located in the materials and methods section that can be run by cutting and pasting it into a Mathematica notebook. The next 5 differential equations account for the voltage-dependent gating variables for Shaker (*nS* and *bb*), Shaw (*n*) and NAV 1.6 (*m* and *h*) (see materials and methods). High voltage-activated Shaw conductance, the delayed rectifier part of the spike generator, was located in compartment 10. Voltage-independent K+ leak conductance, part of which is quickly enabled by Ca++, was distributed evenly amongst all 10 compartments. Low voltage activated Shaker A-type conductance, part of which is also Ca++ sensitive, was placed only into compartment 7 (a model that included 8 compartments with Shaker was slow, but performed similarly). The last two differential equations (below) deal with the second messenger: the dynamical variable *Ca* accounts for Ca++ concentration, which rises with a ∼1 msec time constant and activates K+ leak immediately. *CaS* accounts for a ∼10 millisecond delay for Ca++ to enable Shaker:
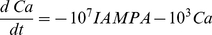
(11)

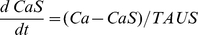
(12)

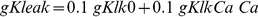
(13)

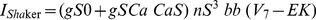
(14)K+ leak conductance *gKleak* has a fixed part *gKlk0* that is not Ca++ sensitive and a part *gKlkCa* that is sensitive to Ca++ concentration. *nS* is Shaker's fast voltage-dependent activation gate (1 msec time constant), *bb* is its slower voltage-dependent inactivation gate (blocking ball with a 3 msec time constant). *gS0* is the amount of Shaker that is not Ca++ sensitive and *gSCa* additional Shaker enabled by Ca++. *V_7_* is the membrane potential in the 7^th^ compartment, and *EK* is the potassium reversal potential (−98 mV). In the second messenger approach AMPA input is assumed to increase Ca++, which then enables more K+ leak and Shaker conductance. After an action potential, and during the return from the after hyperpolarization which follows it, Shaker is quickly activated by its fast voltage sensitive on gates. This slows the rise of membrane potential in between spikes, delaying the next action potential. As Shaker becomes blocked, membrane potential rises faster until a follow on spike is initiated. Shaker “A channels serve as a damper on the interspike interval to space successive action potentials much more widely than a combination of standard Na, K and leak channels could alone” [6].

The dendrite's inward leak H current is noteworthy. At −100 mV, H conductance has two very slow voltage-gating time constants (∼0.5 sec and ∼3.0 sec) that get slower closer to the cell's resting potential [18]. Since the current pulses that we use to represent a tone burst only last for 200 msec, we treat H as just a resistive inward leak. H conductance's weak and slow voltage dependence, combined with its reversal potential's proximity to the membrane potential, imply that its voltage-dependent effect will be much more subtle than would that for a typical voltage-dependent K+ channel. That is, given a small voltage change effecting equal amounts of H and K+ conductance, H would respond ∼100 times more slowly and with ∼10 times less current. It appears likely that H is involved in adaptation effects that occur on longer time scales than those associated with the fast rate-coding of sound intensity. In the first 6 compartments H conductance is placed under simulated feedback control via LOCS fibers (cholinergic efferents from the lateral olive that appear to act through a cAMP second messenger pathway) [14]. We also assume that this part of H is Ca++ sensitive. Thus in the model, parts of K+ leak, Shaker and H are all sensitive to Ca++ concentration, which is itself proportional to input current. This way both the dendrite's fast negative feedback response (K+ leak and Shaker) and its slower LOCS feedback response (H) act proportionally to the input current.

### Simulation Results

A comparison to electronics is interesting. About 40 years ago, engineers at National Semiconductor designed a cheap fast linear amplifier that was composed of 24 intrinsically strongly nonlinear transistors. In its basic (comparator) configuration, their LF411 op amp had two inputs and one output. This output would saturate at the positive power supply voltage (usually +15 volts) when its positive input exceeded its negative input by ∼100 microvolts, and vice versa. In this open loop comparator configuration, the device was strongly nonlinear, with an open loop gain of ∼10^5^, and only about 0.0002 volts of input dynamic range [21]. Hence small hundred micro volt variations in input signal would slew its output rapidly between +15 and −15 volts. However, when employed in one of its negative feedback configurations, the LF411 was intended to be used as a high performance linear amplifier. For example, in a negative feedback configuration called a noninverting amplifier, part of its output voltage was sent back to its minus input, so that its signal gain was enormously reduced, normally down into the times ten range, and its input dynamic range increased to several volts. Here it behaves as a linear amplifier, faithfully duplicating the input signal while stretching it ten-fold.

**Figure 2:**
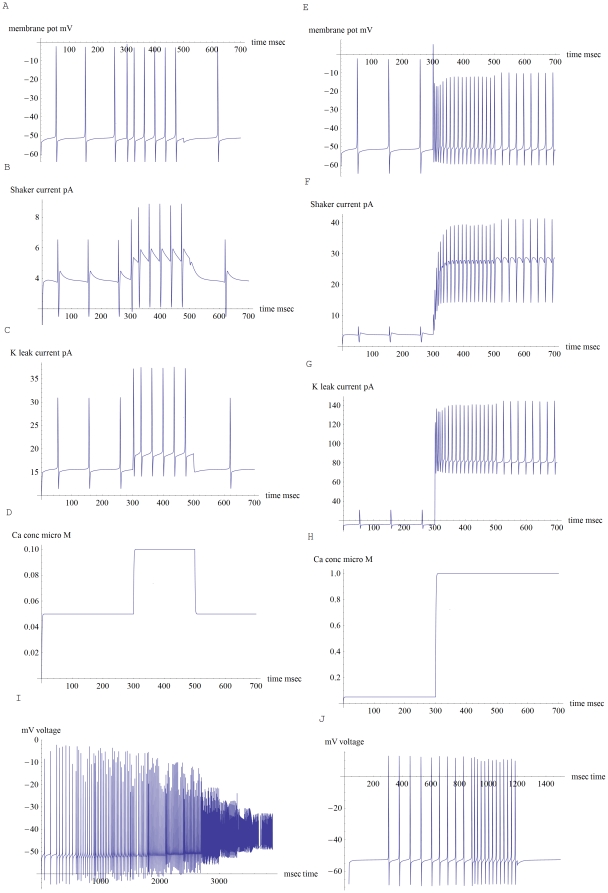
Spike trains, K+ currents and Ca++ concentrations from a model of the distal dendrite, spike generator region of an auditory nerve fiber (ANF). The model employs fast negative feedback to stretch its dynamic range. A. Spike train from a low threshold ANF with 5 pA quiescent AMPA input current. It has a 10 Hz spontaneous rate that is increased to 28 Hz during a 10 pA input that simulates a near threshold tone burst between 300 and 500 msec. B. Outward Shaker K+ current that helps to shape the spike train in part A. Note that Shaker current decreases between spikes. C. Outward K+ leak current for part A. Leak current increases in between spikes. D. Dendritic Ca++ concentration for part A. The second messenger Ca++ was used to initiate negative feedback, turning on K+ leak almost immediately and Shaker with a ∼10 msec delay. E. 100 pA input obtains a 73 Hz spike rate, where the faster rate near start of the pulse is due to the short time delay between input current and outward Shaker current. A part of the Shaker current is due to fast negative feedback that is enabled by Ca++. Also, slower LOCS feedback is assumed to partly turn off H current. To simulate LOCS feedback ∼2 pA of H current was turned off at 500 msec, and this acts to halve the firing rate. F. Outward Shaker current for part E. G. Outward K+ leak current for part E. H. Ca++ concentration for part E. I. Same model without any LOCS feedback is driven by a succession of increasing inputs: 5, 6, 10, 20, 30, 40, 60, 80, 100, 200, 300, 400 and 500 pA. The model fiber has a 40 dB input dynamic range with a maximum spike rate ∼290 Hz. Note that for larger input currents spike amplitude drops due to decreased dendrite impedance. These smaller action potentials could later be enlarged by spike repeaters after the nerve is myelinated [13]. J. High threshold version of the model ANF has a 0 Hz rate for 38 pA input and is driven by the current sequence 38, 39, 40, 50 and 38 pA.

**Figure 3:**
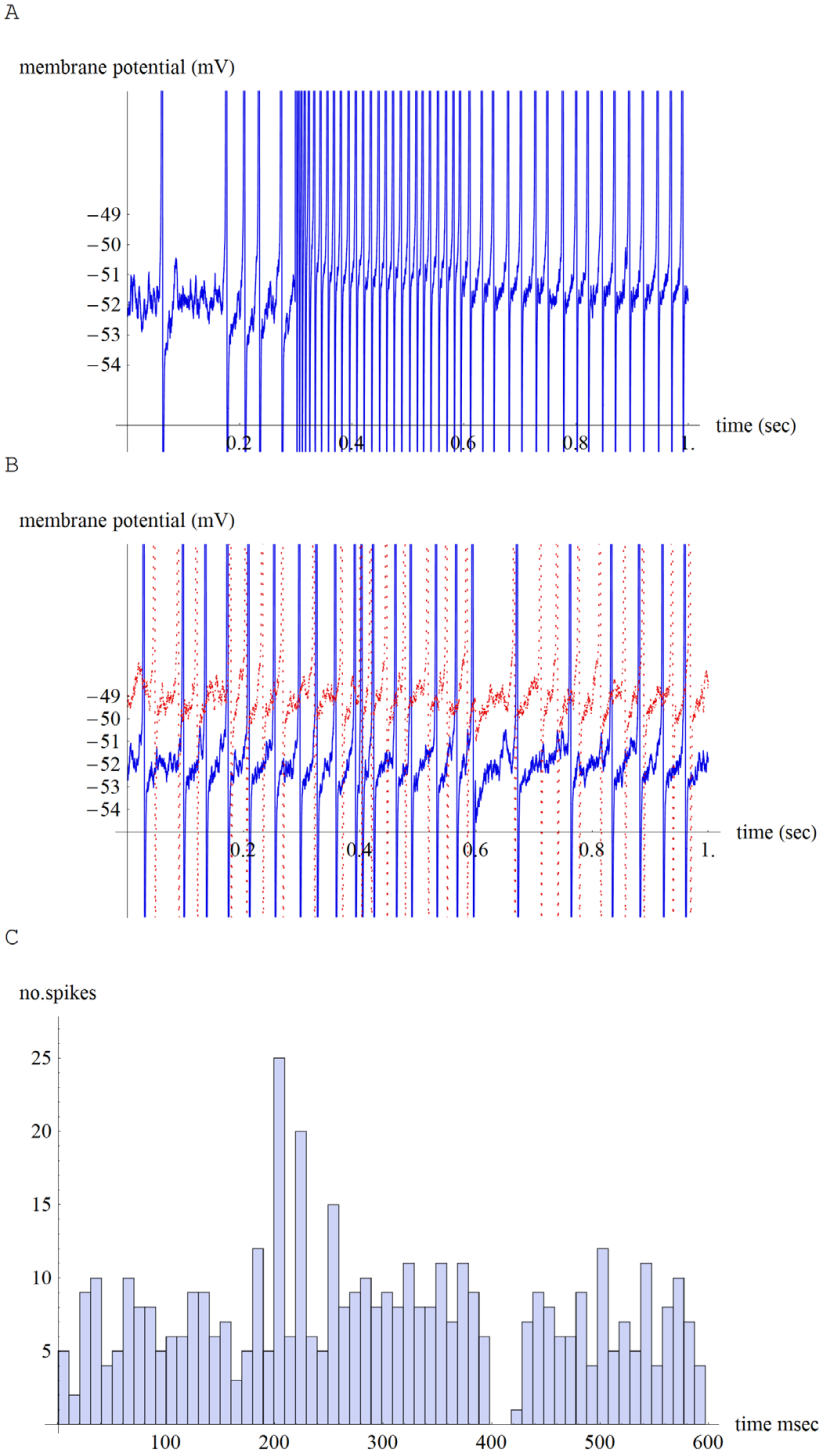
Low threshold model now includes 300 microvolt RMS Johnson voltage noise. A. 100 pA input starting at 0.3 sec obtains a 75 Hz spike rate that is a similar rate to its no noise version in 2 E. Also, similar to 2 E, the spike rate is reduced to 42 Hz by simulating LOCS feedback as turning off H current, starting at 0.6 sec. B. Same low threshold model and noise. Two noisy spike trains each driven by a near threshold 10 pA input current between 0.4 and 0.6 sec (red trace displaced upward by 3 mV). C. Post stimulus time histogram (PSTH) for 30 data sets under the same noise conditions and driven by a 10 pA input between 200 and 400 msec (shown are the cumulative numbers of spikes in sixty 10 msec wide bins). Note the on transient between approximately 200 and 260 msec, the off transient between about 400 and 440 msec and the rate coding of a near threshold current input that occurs between about 260 and 400 msec.

Nature faces a similar problem with spike generators; trying to make an intrinsically nonlinear generator into a linear filter with a low sensitivity threshold and a wide range until it saturates. Fast negative feedback is just one natural way to linearize the output of either an op amp or a neuronal spike generator [21], [9]. As an example, note that a bare spike generator, placed into a model dendrite with a capacitance of 1.5 pF, starts spiking and then saturates at ∼300 Hz, across only about 5 dB of input dynamic range. In Fig. 2 we show noiseless spike trains and currents from two versions of the model dendrite: a low threshold (5 pA) medium spontaneous rate (∼10 Hz) version with a 40 dB dynamic range (Fig. 2 A, E, I), and a high threshold (38 pA) low spontaneous rate version (0 Hz) with a 26 dB dynamic range (Fig. 2 J). Voltage noise will later be added into the low threshold model (Fig. 3). Note that the high threshold model was made less excitable by lowering H conductance (*gH0*) and increasing K+ leak (*gKlk0*). Since this made it more difficult to spike, its spike generator's NAV 1.6 (*gNa*) and high-threshold Shaw conductance (*gK*) were both increased.

**Figure 4:**
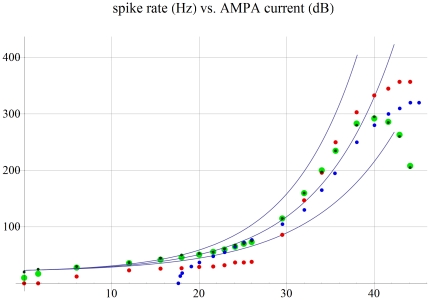
Spike rate versus AMPA input current (0 dB set = 5 pA, or an average of about an EPSC every 16 msec). Comparison is made to linear filter performance for four model variations: large light green dots = low threshold (5 pA) medium spontaneous rate (10 Hz) fiber with a 40 dB dynamic range, red dots = same, but with simulated LOCS feedback that turns off H current, small dark green dots = low threshold model including 300 microvolts RMS of Johnson voltage noise (spontaneous rate increased to ∼20 Hz). Blue dots = high threshold (38 pA) low spontaneous rate (0 Hz) fiber with 26 dB dynamic range. Central line corresponds to spike rate going like the first power of input current, while in the top and bottom lines spike rate goes like 1.1 or 0.9 power, respectively. Fast negative feedback is able to linearize ANF output and to greatly increase its dynamic range. Simulated LOCS feedback that is able to decrease H current is able to increase the number of spikes per dB in the upper intensity range of the low threshold fiber from about 7 to 12, which would increase the information content of auditory spike trains in noisy environments.

In both the low and high threshold cases, fast negative feedback is able to linearize the fiber's f-I curve and greatly extend its dynamic range (Fig. 4). The time delay inherent in such feedback (∼10 msec for Ca++ to turn on Shaker) is able to account for the fast transient spike rate, and subsequent adaptation to a lower rate, that is typically observed when recording from an ANF at the onset of a tone burst [2], [4]. Note that for approximately each 5 pA of net AMPA current input, ∼4.5 pA of outward leak current leaves the fiber, about 30% of it being carried by Shaker (Fig. 2 B, C, F, G). In between spikes, Shaker and K+ leak currents follow opposite trajectories versus time (K+ leak increasing and Shaker decreasing). These currents add together in such a way that the membrane potential's time course becomes well-controlled in between spikes. For example, on a millisecond time scale and millivolt potential scale, membrane potential rises at about a 10 degree angle for 5 pA (quiescent) AMPA current, and at about a 30 degree angle for a 10 pA AMPA input (Fig. 2 A). It rises more steeply, at about a 60 degree angle, for a much larger 100 pA input (Fig. 2 E).

Cholinergic LOCS feedback was simulated to be able to turn off H current, and this effect was able to displace the fiber's dynamic range upwards by ∼26 dB (new range from about 26–43 dB; Fig. 4). For example, for a 100 pA AMPA input, when LOCS feedback turned off ∼2 pA of H current, this cut the spike rate about in half (Fig. 2 E; LOCS feedback shown as occurring instantaneously for the purpose of comparison). Interestingly, H current's low reversal potential in the dendrite (−45 mV), appears to make it ideal for use as a mixed feedback device. For small input currents the average membrane potential is low, and H is excitatory inward leak. However, large input currents raise the dendrite's average membrane potential above −45 mV, so that H becomes outward leak. Hence a LOCS feedback that turns off H, decreases the firing rate for small input currents, and increases it for large ones, while also increasing the saturation current of the ANF. Since all three of these effects increase the information content of auditory spike trains in noisy environments, it would be one good reason for an ANF to have such an unusually low reversal potential for H conductance in its dendrite [6].

There are many different types of noise inside a neuron, but we simulate only simple thermal noise (Fig. 3). In the low threshold model we incorporate 300 microvolts RMS of Johnson voltage noise (*JVN*), as if a thermally-induced white noise spectral density *4 k T R* (in V^2^ per Hz) drives the parallel combination of a 400 M Ohm resistor and a 1.5 pF capacitance across a 10 kHz bandwidth (*k* = Boltzmann's constant, *T* = temperature in degrees Kelvin, *R* = resistance in Ohms, *b* = bandwidth in Hz; 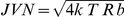) [22]. Such voltage noise injected into compartment 1 of the low threshold model doubles its spontaneous firing rate to about 20 Hz, and the resulting noisy spike train makes a poor fit to a Poisson distribution—mostly because there are insufficient events having short inter spike intervals (ISI; fit mean = 1.28 spikes per 50 msec interval, *r^2^* = 0.73). Note that when fitting spike trains to a spike probability distribution, that we are dealing only with the post synaptic side of the IHC-ANF synapse. That is, we force a high frequency non phase-locking ANF dendrite with a simple square current pulse plus Johnson noise and avoid the complex issue of how noisy vesicle release by the IHC ribbon synapse precisely drives the fiber. Also, note that spontaneous activity in cat ANF does not fit well to a Poisson distribution, but instead fits better to a mix of exponential and gamma distributions [23]. This is due in part to the lack of events having a short ISI [23]. It seems that at least part of the reason for the ANF's anomalously long refractory period, is that after a spike, and in order to make a follow-on spike, the synaptic current charging the spike generator must first pass through an increasingly leaky dendrite.

Compare noisy spike trains to those under the same conditions but without noise (Fig. 3 A with Fig. 2 E and Fig. 3 B with Fig. 2 A). Johnson voltage noise raises the spontaneous and driven firing rates slightly, rendering the f-I curve closer to linear for small inputs (Fig. 4). Fig. 3 B shows two examples of near threshold spike trains. Fig. 3 C shows a post stimulus time histogram (PSTH) for 30 noisy spike trains, each responding to a 200 msec 10 pA input. The PSTH shows how the use of fast negative feedback is able to combine sound timing information (in the onset, offset transients) together with intensity rate-coding information (adapted spike rate in between the transients). For contrast, mechanoreception in some spiders employs pairs of sensory neurons. Type A usually make a single spike that times the onset of the received vibration. Type B neurons make a burst that encodes the vibration intensity [24].

The ear responds to external noise by lowering its gain; feedback from the medial olive (MOCS) lowers the force production of the outer hair cells, which are the active parts of the CA. Electrical stimulation of the MOCS fibers reduces both the amplitude of the oscillation of the basilar membrane, and the spike rates of auditory nerve fibers [25]. MOCS stimulation without background noise lowers ANF minimum firing rate and shifts its sensitivity threshold upwards by ∼10 dB [26]. Moderate noise without MOCS stimulation increases ANF minimum firing rate, and also shifts its sensitivity threshold upwards by ∼10 dB. The two threshold shifts appear to add in a complementary fashion [26].

Why should pairing MOCS feedback to the CA and LOCS feedback to the ANF improve hearing in noisy environments? We use simple information theory to show how simultaneous feedback control of this amplifier-attenuator combination improves the Shannon entropy of the auditory spike trains. Experimental data from Fig. 4 of reference 26 shows that a low threshold high frequency (7.1 kHz) ANF in quiet, increases its spike rate by about 240 Hz across 40 dB of sound pressure input range (∼6 Hz per dB). This drops to ∼4 Hz per dB across ∼43 dB dynamic range in moderate noise, and is subsequently increased to ∼7 Hz per dB across a ∼33 dB range when MOCS feedback is stimulated. Shannon entropy is given by 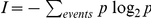 summing over all events where *p* is the probability for a particular event. For this calculation we make the assumption that in the space of natural sounds, intensity goes like a power law: a sound with 10 times more power occurs 10 times less often so that each dB will occur with equal probability. We also assume that the auditory pathway obtains information from a 1 Hz difference in spike rate. Subject to these crude assumptions, each spike rate has an equal probability *p* for occurring, and the Shannon entropy for spike rates in the quiet is 
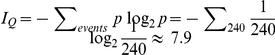
 bits. In this example there are 240 one Hz wide bins each occurring with probability of 1 in 240. In moderate noise, *I_N_*∼7.4 bits, and in noise with MOCS feedback, *I_N+MOCS_*∼7.8 bits. For the low threshold model I∼8.1 bits (Fig. 4). Simulated LOCS feedback is able to stretch the spike rate range above 26 dB by a factor of ∼12/7. So assuming that background noise displaces the sensitivity threshold upwards to 26 dB, and taking the respective dynamic ranges into account (26–40 dB without feedback vs. 26–43 dB with feedback), *I_NO LOCS_*∼6.6 bits while *I_LOCS_*∼7.7 bits. The above simple math arguments support the idea that both MOCS and LOCS feedbacks should work together to optimize the information content of auditory spike trains when background noise is present.

Besides clearly improving signal to noise, what is the advantage of having 20 ANF with different sensitivity thresholds that all originate from a single IHC? The intensity rate code would then be distributed in an intuitively reasonable way amongst 20 noisy parallel spike trains, with the low threshold fibers reporting intensity in a more analog way, and the higher threshold ones perform a sort of digital thresholding (Fig. 4). Note that recently good arguments have been made as to the kinds of auditory filter sets that are most suitable for representing natural sounds [27]. Regarding the choice of thresholds for the high and low threshold model fibers, a mini makes a ∼40 pA EPSC that lasts for about two msec, and a maximal IHC transmitter release is about 800 pA [14], [19]. The high threshold model (38 pA) effectively assumes that almost continuous vesicle release by the IHC is needed to initiate spiking. The low threshold model spikes at ∼10 Hz (∼20 Hz with Johnson noise) with a quiescent 5 pA of net input current, so in Fig. 4 we set 0 dB with respect to this 5 pA current reference. The 5 pA threshold choice amounts to an average of one EPSC every 16 msec, and was made to allow for an approximately hundred fold dynamic range of input currents, that would be consistent with experiment. This way, near threshold, the noisy fiber is able to sense a slight increase in the average rate of IHC vesicle release. Here, fast time-delayed negative feedback makes it advantageous for low threshold fibers to have high spontaneous rates. Then even a small change in input current results in a spike rate transient that is initially magnified before the onset of negative feedback (Figs. 2 A, 3 B). Signal detection close to the sensitivity threshold could then occur by coincidence detection of these high contrast transients between a small number of noisy fibers (Figs. 3 B, C).

## Discussion

Fast negative feedback is able to linearize the ANF f-I curve and stretch its dynamic range. It is unclear whether this feedback should derive directly from a first messenger, the average membrane potential, or from a second messenger such as Ca++, or from some combination of the two. In this paper we use the second messenger approach because using it made it easier to make a rate-code work across a large input dynamic range. However, the first messenger approach does appear to be simpler, and we will examine it in a second paper where we use average membrane potential to generate negative feedback. Meanwhile, here the second messenger approach amounts to the synapse sending a pair of signals, AMPA current and its Ca++ component, and this allows for an extra gating variable on the Shaker channel (Eqn. 14). Note that Shaker is the dominant K+ conductance in ANF [16]. Also note that because the ANF spike generator has a ∼300 Hz spike frequency output range and is located in a small compartment, that its rate-coding task is perhaps the most challenging in the mammalian nervous system. Here Shaker turns on only in a narrow voltage range about 10 mV wide, just below spike threshold, and its fast on and off gates are well suited for making a spike-spacing machine that is able to maintain membrane potential on a well-controlled trajectory in between action potentials (Fig. 2).

AMPA conductance, about 0.4 nS per mini, is important. It implies that large AMPA currents themselves make an intrinsic contribution to the admittance of the dendrite—perhaps up to 9 nS [19]. This would amount to a sort of automatic and immediate negative feedback; increasing the admittance of the fiber by turning on more leak makes it more difficult to spike. Here we consider both Shaker and K+ leak to be turned on by AMPA current. We have avoided the issue of how much of the K+ leak should be considered as intrinsic to the AMPA channels in compartment 1, and how much should be a distributed leak that is activated by a second messenger. Simulations with 80% of the K+ leak confined to compartment 1 give similar results, and it is possible that all of it is simply due to AMPA conductance. However, this sort of leak is insufficient for linearizing the fiber's f-I curve and extending its dynamic range. In the low threshold model, a 500 pA AMPA current enables approximately 15 nS of Shaker (about ¼ of which gets turned on in between action potentials), while also turning on about 7 nS of K+ leak.

It is interesting that the ANF's encoding membrane appears to have been designed with a variable input impedance and a variable space constant in the 100 micron range. As AMPA input current increases, fast negative feedback responds by increasing the dendrite's effective conductance from ∼2.5 to ∼11 nS. Membrane resistance drops from 400 to 100 M Ohm, and the space constant decreases by a factor of two (a large change, considering that it sets the length scale on which membrane potential is exponentially damped). In this way the dendrite's small capacitance (and therefore small admittance at threshold), makes it very sensitive to threshold currents. Fast negative feedback, turning on K+ leak and Shaker, then allows it to readjust its cable properties so as to extend its dynamic range.

Adaptation effects that operate on different time scales are widespread in the auditory pathway. We have avoided adaptation in hair bundles, OHC stiffness, IHC vesicle release, AMPA channel conductance, voltage dependent H current, etc. Instead, we consider only a very fast adaptation that occurs in the encoding step where a short tone burst is quickly translated into a spike train by an ANF. In our simple model fast negative feedback is able to stretch the dynamic range of a high frequency mammalian auditory nerve fiber out to the 40 dB seen in experiments. It linearizes the spike generator's f-I response curve, while at the same time maintaining the fiber's sensitivity. We have also shown that LOCS feedback control of H current is able to displace ANF dynamic range upwards, at the same time stretching its output spike range, both of which would assist MOCS feedback to the cochlear amplifier in improving the information content of auditory spike trains in noisy environments. Essentially the ANF spike generator compartment has been modeled as an attenuator that quickly responds with a lowered gain to increased input to the auditory nerve. The mammalian auditory pathway may have a lot in common with Harold Black's 1928 invention, the negative feedback amplifier, and its ubiquitous modern counterpart, the op amp [21].

## Materials and Methods

Ten compartment Mathematica model of the distal dendrite/spike generator/encoding membrane region of a high frequency non phase-locking mammalian auditory nerve fiber. It uses fast negative feedback to linearize ANF output and extend its dynamic range to ∼40 dB. AMPA input current, via the second messenger Ca++, turns on Shaker and K+ leak currents that linearize the fiber's f-I curve. Parameter values are given for a low threshold (5 pA) medium spontaneous rate (∼10 Hz) model. The high threshold (38 pA) 0 Hz spontaneous rate parameter values are listed in the comments. The model can be run by cutting and pasting it into a Mathematica notebook.

(* parameters *)

AMPA0 = −5.0 10∧−12; (* quiescent AMPA current in Amps *)

AMPA1 = −10.0 10∧−12; (* AMPA input due to increased IHC transmitter release *)

Cm = 1.5 * 10∧−12; (* ANF distal dendrite capacitance in Farads *)

gaxial = 100. 10∧−9; (*axial conductance between dendritic compartments in Siemens *)

gH0 = 1.68 10∧−9; (* 1.30 10∧−9 for high threshold fiber; H conductance in Siemens *)

EH = −.045; (* H current reversal potential in volts *)

gHLOCSCa = 0.0; (* −24.0 10∧−12/(.1 10∧−6); assumes LOCS ACh feedback to H is Ca++ dependent in Siemens per Molar Ca++ concentration; turns off H conductance in the first six compartments *)

Ca0 = 0.0 10∧−6; (* initial Ca++ concentration in Molar *)

gKlk0 = 0.263 10∧−9; (* 0.306 10∧−9 for high threshold fiber; K+ leak conductance that is not Ca++ sensitive *)

gKlkCa = 0.144 10∧−9/(.1 10∧−6); (* 0.130 10∧−9/(.1 10∧−6) for high threshold fiber; Ca++ sensitive K+ leak conductance in S/M *)

EK = −.098; (* K+ reversal potential in volts *)

gK = 5.7 10∧−9; (* 7.0 10∧−9 for high threshold fiber; high threshold Shaw delayed rectifier K+ conductance in S *)

sn = .006; (* sensitivity of activation gate on high threshold Shaw K+ channel in volts *)

TAUn = 0.0013; (* 0.0024 for high threshold fiber; time constant activation gate on high threshold Shaw channel in seconds *)

Vhalfn = −.044; (* half open voltage on activation gate on high threshold Shaw channel *)

gS0 = 0.30 10∧−9; (* non Ca++ sensitive low threshold Shaker conductance in S *)

gSCa = 0.31 10∧−9/(.1 10∧−6); (* Shaker conductance that is Ca++ sensitive in S/M *)

TAUS = .010; (* time constant for Ca++ to turn on Shaker conductance in s *)

snS = .006;(* sensitivity of activation gate on low threshold Shaker channel in volts *)

TAUnS = .001; (* time constant activation gate on low threshold Shaker channel in s *)

VhalfnS = −.062;(*half open voltage activation gate on low threshold Shaker channel *)

sbb = .004; (*sensitivity of blocking ball inactivation gate on Shaker channel in volts *)

TAUbb = .003; (* time constant of inactivation gate Shaker channel in seconds *)

Vhalfbb = −.055;(* half open voltage blocking ball on Shaker in volts *)

gNa = 3.7 10∧−9;(*5.0 10∧−9 for high threshold fiber; max Na+ conductance in Siemens *)

ENa = .067; (* Na+ channel reversal potential in volts *)

sm = .005; (* sensitivity of activation gate on Na+ channel in volts *)

TAUm = .0001; (* time constant of activation gate on Na+ channel in seconds *)

Vhalfm = −.046;(* half open voltage activation gate on Na+ channel *)

sh = .004; (* sensitivity of inactivation blocking ball gate on Na+ channel in volts *)

TAUh = .006; (* activation time constant of blocking ball on Na+ channel in seconds *)

Vhalfh = −.040; (* half open voltage on blocking ball on Na+ channel *)

(* differential equations *)

Eq1 = {−0.1 Cm V1'[t] =  = 

 IAMPA[t]+gaxial (V1[t]−V2[t])+gHfb[t] (V1[t]−EH)+

 gKleak[t] (V1[t]−EK)};

Eq2 = {−0.1 Cm V2'[t] =  = 

 gaxial (V2[t]−V1[t]+V2[t]−V3[t])+

 gHfb[t] (V2[t]−EH)+gKleak[t] (V2[t]−EK)};

Eq3 = {−0.1 Cm V3'[t] =  = 

 gaxial (V3[t]−V2[t]+V3[t]−V4[t])+

 gHfb[t] (V3[t]−EH)+gKleak[t] (V3[t]−EK)};

Eq4 = {−0.1 Cm V4'[t] =  = 

 gaxial (V4[t]−V3[t]+V4[t]−V5[t])+

 gHfb[t] (V4[t]−EH)+gKleak[t] (V4[t]−EK)};

Eq5 = {−0.1 Cm V5'[t] =  = 

 gaxial (V5[t]−V4[t]+V5[t]−V6[t])+

 gHfb[t] (V5[t]−EH)+gKleak[t] (V5[t]−EK)};

Eq6 = {−0.1 Cm V6'[t] =  = 

 gaxial (V6[t]−V5[t]+V6[t]−V7[t])+

 gHfb[t] (V6[t]−EH)+gKleak[t] (V6[t]−EK)};

Eq7 = {−0.1 Cm V7'[t] =  = 

 gaxial (V7[t]−V6[t]+V7[t]−V8[t])+

 0.1 gH0 (V7[t]−EH)+gKleak[t] (V7[t]−EK)+

 IShaker[nS]};

Eq8 = {−0.1 Cm V8'[t] =  = 

 axial (V8[t]−V7[t]+V8[t]−V9[t])+

 0.1 gH0 (V8[t]−EH)+gKleak[t] (V8[t]−EK)};

Eq9 = {−0.1 Cm V9'[t] =  = 

 axial (V9[t]−V8[t]+V9[t]−V10[t])+

 0.1 gH0 (V9[t]−EH)+gKleak[t] (V9[t]−EK)};

Eq10 = {−0.1 Cm V10'[t] =  = 

 axial (V10[t]−V9[t])+0.1 gH0 (V10[t]−EH)+

 gKleak[t] (V10[t]−EK)+INav[m] + IShaw[n] };

Eq11 = {nS'[t] =  = (nSinf[V7]−nS[t])/TAUnS};

Eq12 = {n'[t] =  = (ninf[V10]−n[t])/TAUn};

Eq13 = {bb'[t] =  = (bbinf[V7]−bb[t])/TAUbb};

Eq14 = {m'[t] =  = (minf[V10]−m[t])/TAUm};

Eq15 = {h'[t] =  = (hinf[V10]−h[t])/TAUh};

Eq16 = {Ca'[t] =  = −10∧7 IAMPA[t]−10∧3 Ca[t]}; (* effectively a 1 msec time constant for Ca++ turning on K+ leak *)

Eq17 = {CaS'[t] =  = (Ca[t]−CaS[t])/TAUS}; (* slower 10 msec time constant for Ca++ to turn on Shaker *)

(* auxiliary equations *)

IAMPA[t_]: = If[t>0.3 && t<0.5, AMPA1, AMPA0];

IShaker[nS_]: = (gS0+gSCa CaS[t]) nS[t]∧3 bb[t] (V7[t]−EK);

nSinf[V7_]: = 1./(1.+Exp[(−V7[t]+VhalfnS)/snS]);

bbinf[V7_]: = 1./(1.+Exp[(V7[t]−Vhalfbb)/sbb]);

INav[m_]: = gNa m[t]∧3 h[t] (V10[t]−ENa);

minf[V10_]: = 1./(1.+Exp[(−V10[t]+Vhalfm)/sm]);

hinf[V10_]: = 1./(1.+Exp[(V10[t]−Vhalfh)/sh]);

IShaw[n_]: = gK n[t]∧3 (V10[t]−EK);

ninf[V10_]: = 1./(1.+Exp[(−V10[t]+Vhalfn)/sn]);

gHfb[t_]: = 0.1 gH0+If[t>.5, gHLOCSCa/6. Ca[t], 0];

gKleak[t_]: = 0.1 gKlk0+0.1 gKlkCa Ca[t];

(* solve the system *)

InitCond = {V1[0] =  = −0.060, V2[0] =  = −0.060, V3[0] =  = −0.060,

 V4[0] =  = −0.060, V5[0] =  = −0.060,

 V6[0] =  = −0.060, V7[0] =  = −0.060, V8[0] =  = −0.060, V9[0] =  = −0.060,

 V10[0] =  = −0.060, m[0] =  = 0.0, h[0] =  = 0.0, n[0] =  = 0.5,

 nS[0] =  = 0.5, bb[0] =  = 0.5, Ca[0] =  = Ca0, CaS[0] =  = Ca0};

Eqns = Join[Eq1, Eq2, Eq3, Eq4, Eq5, Eq6, Eq7, Eq8, Eq9, Eq10, Eq11,

 Eq12, Eq13, Eq14, Eq15, Eq16, Eq17, InitCond];

Res = NDSolve[Eqns, {V1, V2, V3, V4, V5, V6, V7, V8, V9, V10, m, h, n, nS, bb,

 Ca, CaS}, {t, 0.0, 0.7}, MaxSteps→1000000000, AccuracyGoal→14];

(* output plot *)

Plot[1000*V10[t/1000]/. Res, {t, 0, 700}, AxesLabel→{time msec, voltage mV}, PlotRange→All]
